# Immunoaffinity Capillary Electrophoresis in the Era of Proteoforms, Liquid Biopsy and Preventive Medicine: A Potential Impact in the Diagnosis and Monitoring of Disease Progression

**DOI:** 10.3390/biom11101443

**Published:** 2021-10-01

**Authors:** Norberto A. Guzman, Daniel E. Guzman

**Affiliations:** 1 Princeton Biochemicals, Inc., Princeton, NJ 08543, USA; daniel.guzman@ucsf.edu; 2 Division of Hospital Medicine, Department of Medicine, University of California at San Francisco, San Francisco, CA 94143, USA

**Keywords:** proteoforms, isoforms, low abundance biomarkers, liquid biopsy, immunoaffinity capillary electrophoresis, molecular biorecognition, point-of-care instrument, proteomics, precision medicine, preventive medicine, cancer, telemedicine, exosomes, circulating tumor cells, circulating immunological cells

## Abstract

Over the years, multiple biomarkers have been used to aid in disease screening, diagnosis, prognosis, and response to therapy. As of late, protein biomarkers are gaining strength in their role for early disease diagnosis and prognosis in part due to the advancements in identification and characterization of a distinct functional pool of proteins known as proteoforms. Proteoforms are defined as all of the different molecular forms of a protein derived from a single gene caused by genetic variations, alternative spliced RNA transcripts and post-translational modifications. Monitoring the structural changes of each proteoform of a particular protein is essential to elucidate the complex molecular mechanisms that guide the course of disease. Clinical proteomics therefore holds the potential to offer further insight into disease pathology, progression, and prevention. Nevertheless, more technologically advanced diagnostic methods are needed to improve the reliability and clinical applicability of proteomics in preventive medicine. In this manuscript, we review the use of immunoaffinity capillary electrophoresis (IACE) as an emerging powerful diagnostic tool to isolate, separate, detect and characterize proteoform biomarkers obtained from liquid biopsy. IACE is an affinity capture-separation technology capable of isolating, concentrating and analyzing a wide range of biomarkers present in biological fluids. Isolation and concentration of target analytes is accomplished through binding to one or more biorecognition affinity ligands immobilized to a solid support, while separation and analysis are achieved by high-resolution capillary electrophoresis (CE) coupled to one or more detectors. IACE has the potential to generate rapid results with significant accuracy, leading to reliability and reproducibility in diagnosing and monitoring disease. Additionally, IACE has the capability of monitoring the efficacy of therapeutic agents by quantifying companion and complementary protein biomarkers. With advancements in telemedicine and artificial intelligence, the implementation of proteoform biomarker detection and analysis may significantly improve our capacity to identify medical conditions early and intervene in ways that improve health outcomes for individuals and populations.

## 1. Introduction

Clinical medicine is currently practiced based on the evaluation of symptoms and signs of disease with diagnostic tests and the lack thereof with a handful of screening tests; the results of these tests help to determine what illness a patient has. Ultimately, identifying a disease and determining its severity leads to targeted therapies and lifestyle interventions, with the hope that a patient’s symptoms and signs of disease will improve [1,2,3,4]. The implication of a clinical structure that primarily functions on disease presentation is that most of healthcare spending is focused on treating rather than preventing chronic disease [5,6]. Based on the Centers for Medicare and Medicaid Services (CMS), the United States spends approximately 75% of annual healthcare spending on treating chronic disease alone [5,6]. Despite this, chronic diseases, such as heart disease, stroke, cancer, chronic kidney disease, and diabetes continue to be the leading causes of death, disability, and reduced quality of life in the United States [7,8]. Such a focus on treatment of illness once it clinically manifests has brought into question how improvements in and implementation of preventive medicine could reduce the healthcare burden on patients and the healthcare system as a whole.

For decades, we have known that disease manifests before it is clinically apparent or, in other words, before it manifests with symptoms and signs recognizable to patients and their health providers [9,10,11]. Many chronic diseases are “silent” and may take several years to manifest [12,13,14,15,16,17,18]. In 1999, for example, a large prospective study on Type 2 Diabetes Mellitus published in the United Kingdom demonstrated that pancreatic beta-cell dysfunction starts almost a decade before patients are diagnosed with Type 2 Diabetes Mellitus [19,20]. The consequence of this based on current testing is that patients will have already lost up to 50% of their beta cell function by the time practitioners are able to diagnose them with this disease, making treatment and cure of the disease more difficult [19,20,21,22,23]. To give another example, in a study of the evolutionary history of 2658 cancers, researchers determined that there are genetic mutations that occur far before the clinical manifestation of cancers that can be detected, if tested for [24]. For instance, the rate of cytosine guanine (CpG) to thymine guanine (TpG) mutations can be a surrogate to predict latency of presentation of disease, with ovarian adenocarcinoma presenting up to 30 years after the occurrence of these mutations [24]. It has therefore become apparent that a stronger emphasis on screening for disease and disease monitoring, coupled with early targeted therapeutic interventions can ultimately prevent the manifestation and burden of symptoms and signs of disease.

**Figure 1:**
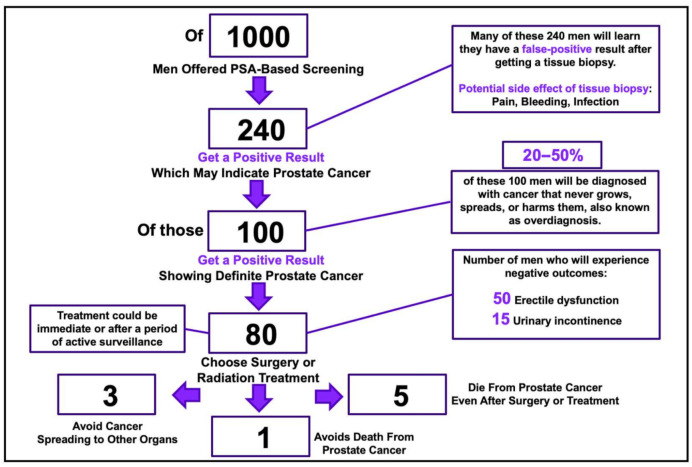
Depicts a schematic published from the USPSTF in 2018, detailing the use of PSA for prostate cancer screening. Figure modified from [29].

While diagnostic testing methods have advanced in many ways over the past several decades, detection of biomarkers prior to or in the early stages of disease manifestation remains limited [25,26]. A handful of screening tests have been approved for diseases such as colon cancer, breast cancer, prostate cancer, and lung cancer, however most of these tests utilize imaging modalities and direct visualization with only colon cancer and prostate cancer offering laboratory diagnostic detection. The majority of laboratory diagnostic testing is now carried out via immunoassays [27], which have proved reliable if the clinical suspicion for disease is moderate to high. Nevertheless, current immunoassays remain limited when applied to early detection of disease due to polyreactivity of their antibodies, the presence of interfering substances in a sample, and concentrations of biomarkers below levels of detection [27,28]. For example, use of existing immunoassays to detect prostate specific antigen (PSA) for prostate cancer screening is controversial due to varying accuracy of results (see Figure 1).

In summary, the above schematic demonstrates that for every 1000 men screened for prostate cancer, only 1 will avoid death from prostate cancer and 3 will avoid metastatic spread of the cancer, with the remaining men suffering psychological harm of a potential diagnosis of cancer, risks of prostate biopsy, and side effects of erectile dysfunction and urinary incontinence from treatment of prostate cancer [29]. While there is no clear evidence to recommend for or against prostate cancer screening, it is clear that the potential harm from screening may outweigh the potential benefit for many individuals due to false positives and a lack of understanding of the severity of different prostate cancers.

In theory, elimination of false-positive and false-negative results from immunoassays could result in greater reliance on laboratory testing, particularly in the use of screening and monitoring for a variety of diseases without the need for clinical interpretation [30,31]. Ultimately, such a change could revolutionize the way clinical medicine is practiced and create a stronger emphasis and investment in prevention of disease through accurate screening tests. The goal of information gathering in the diagnostic process is to reduce the diagnostic uncertainty enough to make optimal decisions for subsequent care [32]. Profiling of the genome and transcriptome are now established tools for the discovery of novel biomarkers, but alterations in proteome expression are more likely to reflect changes in disease pathophysiology [33]. We will therefore discuss advancements in the field of proteomics and how a useful technology known as immunoaffinity capillary electrophoresis can potentially overcome limitations in sensitivity, specificity, and predictive value seen in current immunoassays.

## 2. Proteoforms as Diagnostic Biomarkers

**Figure 2:**
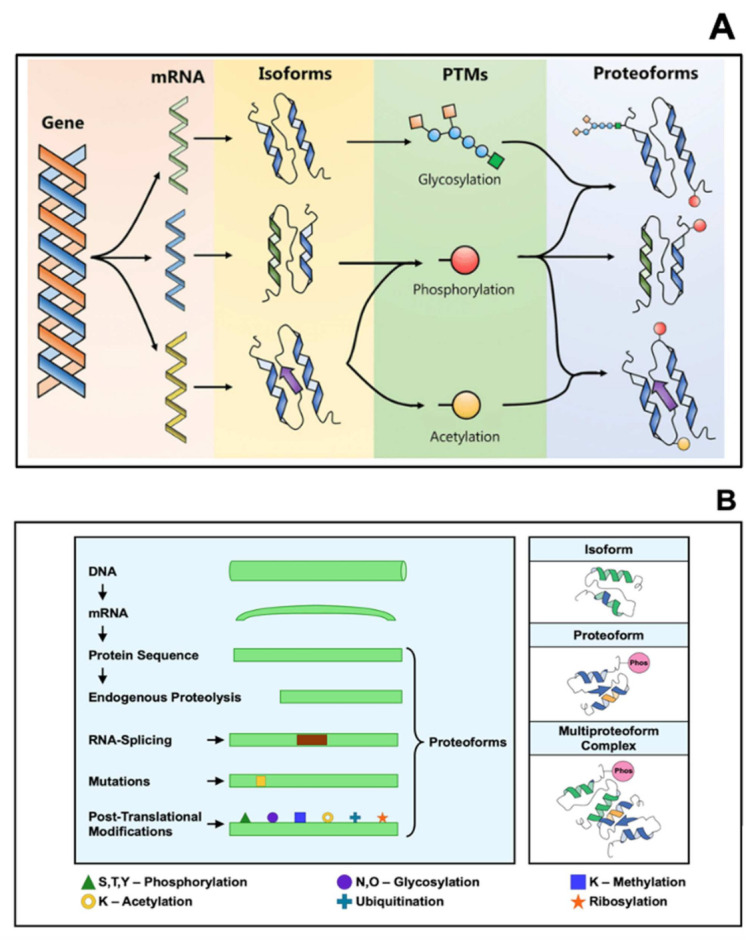
Schematic representation of (**A**) a single gene and three isoforms; the isoform variations combine with site-specific changes to generate proteoforms via post-translational modifications (PTMs). (**B**) Schematic depicting various types of isoforms, including individual examples of an isoform, a proteoform, and a multiproteoform complex. Figures modified from [39,43].

Proteins are the true machines of life and are considered to be the “workhorses” of the cell [34]. They provide functional context to cells, tissues, and organisms in the form of receptors, signaling cascades, channels, and transporters. Proteins are also major structural components of the cytoskeleton and extracellular matrices [35]. They are uniquely identified by their proteoforms, defined as different forms of a protein derived from a single gene including all forms of genetic variations (e.g., amino acid variation), alternative splicing, and post-translational modifications (PTM) [36,37,38,39]. This means that one transcribed gene can lead to a variety of protein structures, and that the biological function of each proteoform, as well as the cellular location, binding partners and kinetics can vary greatly [40] (Figure 2). As a consequence of the vast number of proteoforms that can be formed, the number of proteins in an individual’s proteome far exceeds the number of protein-coding genes in the human genome [41,42]. The identification of more proteoforms associated with different diseases will undoubtedly be an essential landmark discovery necessary for early diagnosis, prevention and treatment of disease [43,44].

While proteomics, or the study of proteins, has become an increasingly important topic of study amongst researchers, proteins and enzymes biomarkers have been used as diagnostic tools for many years [45,46,47]. This is because disruptions of the highly orchestrated actions of proteins is the cause of practically all diseases. Examples of proteoforms that have already had a large clinical impact are apolipoprotein proteoforms, *β*-type natriuretic peptide (*β*NP), proteins found in disorders of glycosylation, detection of structural changes in transthyretin, hemoglobin proteoforms, cystatin C-truncated proteoforms, C-reactive protein, vitamin D-binding protein, transferrin, and immunoglobulin G (NISTmAb) (See Table 1) [48,49,50,51,52,53,54,55,56,57,58,59,60,61,62,63,64,65,66,67,68,69,70,71,72,73,74,75,76,77,78,79,80,81,82,83,84,85,86,87,88,89,90,91,92,93,94,95,96,97,98,99,100,101]. Ultimately, analysis of the human proteome, can give further insight into the modifications and dynamic intermolecular collisions that proteins undergo in disease states [102,103].

Posttranslational modifications (PTMs) of proteins can happen at any step of the protein lifespan in both physiological and pathophysiological states. However, aberrant states of PTMs are frequently implicated in diseases [102,104,105]. Some of the most common protein modifications are hydroxylation, phosphorylation, acetylation, methylation, glycosylation, deamidation, prenylation, ubiquitylation, sumoylation, and proteolysis, among many others [104]. In particular, proteolysis generates protein isoforms or proteoforms with function and properties distinct from their precursors [105,106]. These proteolytic proteoforms regulate various biological processes and act as hormones, neurotransmitters, and disease pathogens. Unlike signal peptide or prodomain removal, however, protease-generated proteoforms can rarely be predicted from gene sequences [106]. Proteolytic processing is therefore a pervasive and irreversible post-translational modification that expands the protein universe by generating new proteoforms [106]. Deregulated proteolysis is a key driver for human diseases, and proteases are promising targets for tailored therapeutics [105,107].

Alterations in proteolytic systems underlie multiple pathological conditions [108]. For example, when viruses such as the SARS-CoV-2 virus infect a host, several proteolytic enzymes, either of the host or the virus, act in a concerted fashion to regulate and coordinate specific steps of viral replication and assembly [109]. To mitigate the high rate of infectivity of SARS-CoV-2, researchers have suggested that targeting host proteases involved in the entry of the viral genome can act as a potential means to block the ability of the virus to infect host cells [109,110]. Clinicians have even suggested the use of a clinically approved protease inhibitor as a potential treatment option for those infected with SARS-CoV-2 [111]. An advantage of targeting host proteases to interfere with enveloped-virus entry is that this mechanism is common to several viruses and therefore may be applied to the treatment and prevention of infection with future novel viruses [109].

In another example, matrix metalloproteinases, a class of metal-linked zinc-dependent proteases that cleave internal peptide bonds of proteins to degrade extracellular matrices, have been linked to the development of cardiovascular disease in obese individuals [112]. For example, MMP-2 activity has been noted to be increased in obese rats with spontaneous hypertensive heart failure [113]. By targeting MMP directly through pharmacological inhibition, researchers found improved left ventricular function and reduced cardiac remodeling, thus suggesting that MMP activity contributed to left ventricle dysfunction in these obese rats [113]. This correlation has been seen in obese woman and adolescents as well, thereby demonstrating that direct inhibition of matrix metalloproteinases may potentially be used in the treatment of heart failure with reduced left ventricular function in obese individuals [114,115].

While the identification and inhibition of many proteases and proteoforms have demonstrated to be clinically useful in hampering disease development and progression, detecting these proteins through existing laboratory methods can be extremely challenging.

## 3. Limitations of Existing Immunoassays Used in Clinical Diagnostics

Immunoassays are one of the most widely used detection methods for detecting protein biomarkers in clinical diagnostics, biopharmaceutical analysis, environmental monitoring, and food testing [116]. For most part, enzyme-linked immunosorbent assay (ELISA), immunohistochemistry (IHC) and flow cytometer assays in particular are immunoassays routinely found in diagnostic laboratories. ELISA continues to be the gold standard for biomarker detection [117,118,119,120,121,122,123] because of its high precision, throughput, and sensitivity (In clinical diagnostic chemistry, a sensitive test refers to a test that will correctly identify almost all individuals who likely have a disease, and will rarely yield a false negative result. A specific test refers to a test that will almost always correctly rule out those who do not have a disease, and will rarely yield a false positive result [117]. In analytical chemistry, sensitivity is the minimal detectable concentration of an analyte, expressed as the limit of detection (LoD), that can be accurately measured.). While the last two decades has seen tremendous innovation in ELISA technology, including automation with robotic workstations, improved microtiter plate readers, higher throughput well formats, and better antibody immobilization techniques, it has not been able to overcome for several reasons [119,120,121,122,123] limitations of diagnostic sensitivity[117] and specificity (Specificity is the ability to accurately assess a single analyte in the presence of components which may be expected to be present in the sample matrix, such as interferences. Similar to specificity, selectivity is the ability to accurately separate out and assess multiple components present in a sample mixture or matrix [119]).

**Figure 3:**
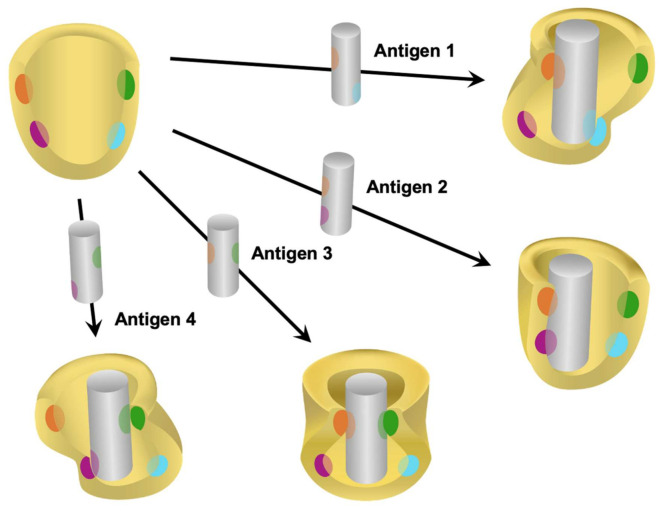
Schematic diagram showing the conformation hypothesis explaining polyreactivity. The classic ‘lock and key’ model of antigen-antibody interaction proposes rigid structures of interacting components and has been used for years to explain the monoreactivity of antibodies. Recent studies suggest that the antigen-binding ‘pocket’ of many antibody molecules is more flexible than previously thought and thus accommodate different antigenic configurations. Figure 3 shows four different antigens interacting with different amino acid residues within the antigen-binding pocket of a single broadly polyreactive antibody molecule. Each interaction alters the folding or conformation of the antigen-binding pocket in a different way. Figure modified from [28].

First, ELISA and other immunoassays are prone to generate false positive results due to the polyreactivity of antibodies and antibody-like molecules. In theory, ELISA uses monoclonal antibodies that should provide higher specificity than polyclonal antibodies because they bind to a single epitope and usually have high affinity. In practice, there are several cases in which monoclonal antibodies have polyreactivity (Figure 3) [28,124,125,126,127,128]. One of the most prominent causes of antibody cross-reactivity or multi-specificity is molecular mimicry. Molecular mimicry is structural, functional or immunological similarities shared between macromolecules found in infectious pathogens and in host tissues [129,130,131,132]. Therefore, a false positive can result from binding of a substance different from the one of interest due to structural similarity in its antigens.

In addition to the polyreactivity of antibodies that are coated to the surface of a well that leads to binding of incorrect analytes of interest, interfering substances are another reason for inaccuracy of results in ELISA [133,134]. An interfering substance is one that remains in a test sample, after the washing steps in ELISA, and is able to bind a detection antibody and can elicit an enzyme-substrate reaction, thus producing a false positive result. Interfering substances include both endogenous substances, such as heterophile antibodies and binding proteins, and exogenous substances, such as antibodies administered to a patient for immunotherapy [133,134,135,136,137,138]. An example of an interference substance causing a false-positive result is a study of 12 women who were diagnosed with postgestational choriocarcinoma on the basis of persistently positive human chorionic gonadotropin (hCG) test results in the absence of pregnancy [139]. In one of these cases, a young woman underwent a hysterectomy, chemotherapy, radiotherapy, and a partial pneumonectomy before the interference was discovered and her diagnosis was corrected [139,140].

Lastly, antibodies used in ELISA can have difficulty binding biomarkers of interest when a signature protein is in the presence of other endogenous proteins where the concentration in some cases is a billion (1 × 10^12^) times higher [141]. Such a dilutional factor makes it extremely difficult for a binding interaction to occur, thus resulting in a false-negative result. While this may seem trivial, crucial proteins with diagnostic value are found in the very low abundance range (<10^−6^ g/L) in biological fluids [141,142,143].

In order to improve clinical utility in detecting and monitoring proteins, existing testing must therefore progress in ways that allow for enhanced sensitivity, to overcome antibody polyreactivity and interference substances, and robust quantification of biomarkers, to detect those found in low concentration levels in biological samples. One valuable strategy to do this is to employ capillary electrophoresis, a reliable separation technique, with affinity-capture.

## 4. Capillary Electrophoresis as a Useful Technology for the Separation of Structurally Related Small Molecular-Weight Substances and Biomolecules

Capillary electrophoresis (CE) is a family of electrokinetic separation methods performed in submillimiter capillaries (conventional capillary electrophoresis) and in micro and nanofluidic channels (microchip electrophoresis) filled with a conductive fluid of a certain pH value. The basic principle of capillary electrophoresis is the separation of ions based on their electrophoretic mobility (mass/charge ratio) with the use of an applied voltage. Capillary electrophoresis can separate a wide range of chemical and biochemical entities, including neutral substances, cellular and subcellular entities, and nanoparticles or nanovesicles using one or more of 9 different separation modes [144,145,146,147,148,149,150,151]. Because of this, CE is known for its high-power resolution, high sensitivity and rapidity, high peak capacity, high separation efficiency with reproducible quantification, the use of small sample volumes with low waste generation, on-column and off-column detection, automation, low cost of operation, use of miniaturized components, option of multiple detection systems, and a diverse range of applications [144,145,146,147,148,149,150,151]. Compared to many other laboratory techniques, capillary electrophoresis can discern minor changes in substances that are structurally very similar.

In general, capillary electrophoresis in conventional or microchip format offers practical as well as technical advantages over traditional electrophoresis and other chromatographic techniques. When using narrow capillary tubes, the lateral diffusion effects are reduced, and the temperature differences across the tubes are also reduced. Furthermore, using narrow capillaries helps to reduce band-broadening seen in the peaks generated in other techniques such as high-performance liquid chromatography (HPLC). As a result, separation efficiency in CE is very high [144,145,146,147,148,149,150,151]. As opposed to conventional electrophoresis, which is labor-intensive and can take several hours to perform, CE can be completely automated and only a few microliters of sample are needed for an analysis to be carried out in a few minutes [123,151]. Although some commercial instruments are quite expensive, there are now many possibilities to manufacture miniaturized laboratory-made instruments for less than $5000 with inexpensive components readily available [123,152]. Reagents and consumables are in general also inexpensive since minute amounts of reagents, buffers and solvents are used than traditional techniques. When performing molecular genotyping, CE detected more alleles and provided higher discriminatory power in comparative studies with agarose gel electrophoresis [153]. Regarding sodium dodecyl sulfate-polyacrylamide gel electrophoresis, a classical technique used for more than four decades but is time-consuming and labor intensive, CE shows many advantages such as on-column detection, automated operation, great resolving power, and capability of accurate protein quantification and molecular weight determination [154]. Regarding the throughput of sample analysis, most commercial analytical separation instruments, including HPLC and CE, are equipped to operate with one column making the process a low-throughput system. A recent report [89] comparing HPLC to CE for the analysis of glycans, were able to demonstrate using a CE ABI 3500 instrument (Applied Biosystems-Thermo Fisher) a high throughput for CE analysis. While the HPLC took as long as an hour to run a single sample, the ABI instrument was able to analyze 24 samples simultaneously in the span of approximately 15 min, with the ability to load two 96 well plates simultaneously, thus enabling the processing of as many as one thousand samples in a day. In addition to its remarkable throughput, this approach demonstrates robust sensitivity, permitting full glycan analysis with small quantities of high-value samples, requiring as little as 1 uL of plasma [89]. If IACE were to be incorporated in sample preparation prior to sample introduction into the CE system, the entire analysis system could be automated and faster.

**Figure 4:**
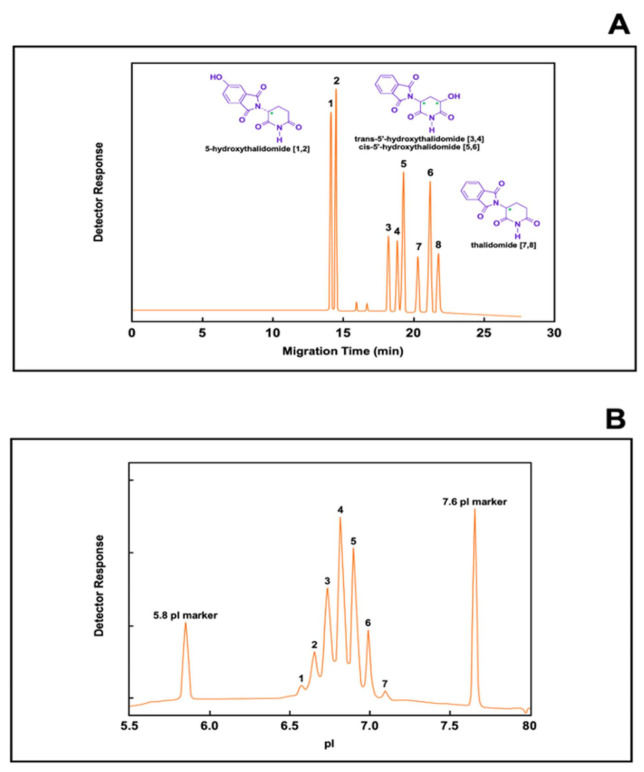
Depicts the power of resolution of capillary electrophoresis. (**A**) depicts an electropherogram showing the simultaneous separation of thalidomide and its hydroxylated metabolites. In this case, the separation of thalidomide and its metabolites was performed by chiral capillary chromatography (CCC) using a single point detection at the outlet end of the capillary. (**B**) depicts an electropherogram showing the various charged isoforms of a recombinant monoclonal antibody. In this case, the separation of the antibody isoforms was performed by capillary isoelectric focusing using a detection system that covers the entire capillary, known as imaged capillary isoelectric focusing (iCIEF) containing the pI markers 5.8 and 7.6. Figures modified from [159,164].

Typical examples of CE electropherograms for small molecular weight substances and a biomolecule are presented in Figure 4. The electropherogram, or plot of detector signal over time, depicted in Figure 4A shows the simultaneous separation of thalidomide and its hydroxylated metabolites by chiral capillary chromatography (CCC), a mode of capillary electrophoresis separation for the separation of enantiomers [155,156,157,158]. The prerequisite of separation of enantiomers in CE is their enantioselective interaction with a chiral selector. Cyclodextrins have the shape of a torus with a hydrophobic interior and a hydrophilic outside. The hydrophilic external shell allows dissolution in the background electrolyte, whereas the lipophilic cavity is responsible for host–guest type interactions leading to the formation of inclusion complexes. In essence, each enantiomer will have a different binding (affinity) constant to the chiral selector, resulting in different effective mobilities and the ability to separate structurally related compounds [159]. In the example presented in Figure 4, in order to achieve optimal separation, the background electrolyte or running buffer contains two cyclodextrins as the chiral selector.

Figure 4B shows an electropherogram of a therapeutic monoclonal antibody. The electropherogram depicted in Figure 4B shows the different charge heterogeneity of the monoclonal antibody separated by imaged capillary isoelectric focusing (iCIEF) [160,161,162,163], a system in which the entire capillary is monitored by the whole-column detection system. An advantage of this technique over conventional CIEF is that mobilization of the protein zones is not required; therefore, the various charged variants of an antibody sample can be simultaneously recorded by the whole-column detector without disturbing the separation resolution [164].

As shown in Figure 4A,B, the electropherograms provides only separation and quantification of each entity represented by a peak intensity or peak area. However, partial structural information of each peak can be achieved by using the proper known standards employing the same experimental conditions. Conversely, a more complete structural analysis is accomplished by coupling CE to a mass spectrometer, a method capable of measuring mass-to-charge ratios of ions using a single instrument (MS) or in tandem (MS/MS), thereby increasing the ability to analyze chemical samples.

Although the separation of molecules using the various modes of capillary electrophoresis described above can achieve high resolution of a wide range of analytes in a short analysis time, the limits of detection (LOD) are constrained by the small sample volumes (nanoliters) introduced into the capillaries and the small dimensions (micrometers) of the capillaries. The shorten pathlength of the capillary hinders common optical detection methods, such as UV detection [151,165,166,167,168]. Usually, optimal electropherograms can be obtained when using one or more of the 9 separation modes of CE if the target analytes are introduced into the capillary at high concentrations. However, working with diluted samples or complex mixtures where the target analytes are found at subnanomolar concentrations poses a significant limitation for detection of these analytes via CE; overcoming the poor sensitivity of CE has remained to be a challenge for many investigators [123,169,170,171,172,173,174,175,176,177,178,179,180]. We will therefore discuss immunoaffinity capillary electrophoresis, a method of CE that prevail over many of these limitations. If IACE were to be incorporated in sample preparation prior to sample introduction into the capillary or microchip, the entire system could be automated, reduce analysis time, and overcome limitations in poor detection sensitivity. We will emphasize the advantages for the determination of proteoforms when compared with other methods described to enhance the analytical sensitivity of analytes, such as field-amplified sample stacking, large-volume sample stacking, pH-mediated stacking, isotachophoreris (ITP), stacking techniques in micellar electrokinetic chromatography (MEKC), soli-phase extraction, and tagging target analytes with various chromophores and detecting the derivatized substances with sensitive detectors [123,151,152,173,174,175,176,177,178,179,180,181,182,183,184,185,186,187,188,189,190]. In some instances, IACE can be used in combination with other methods to enhance analytical sensitivity even further.

## 5. Advantages of Immunoaffinity Capillary Electrophoresis for the Detection of Proteoforms

Immunoaffinity capillary electrophoresis (IACE) is one of the three methods under the classification of affinity capillary electrophoresis. In general, affinity capillary electrophoresis is a broad term referring to the separation by capillary electrophoresis of substances that participate in specific or non-specific noncovalent affinity interactions during electrophoresis. The interacting molecules can be found in solution or can be immobilized to a solid support [123,146,174,175,176,177,178,179,180,191,192,193,194,195,196,197,198,199,200,201,202,203]. Immunoaffinity capillary electrophoresis, is the method referred to when using immobilized affinity ligands or probes to capture one or more analytes found in simple or complex matrices. Once captured in a portion of the capillary, or in a channel in microchip electrophoresis, the target analyte is isolated by eliminating the remaining sample, then releasing it from the immobilized ligand using an appropriate eluting buffer or solution. As a result, the target analyte is purified from the matrix and concentrated, significantly enhancing its limits of detection. To avoid confusion when referring to affinity capillary electrophoresis, we have defined the terminology as free-solution affinity capillary electrophoresis (FSACE) to the two methods of affinity capillary electrophoresis whose interactions are carried out in solution, and immobilized affinity capillary electrophoresis or immunoaffinity capillary electrophoresis (IACE) when the ligand or probes are immobilized to a surface within a portion of the capillary or channel. However, IACE not only uses antibodies or antibody fragments as highly selective capture agents, but also other biorecognition affinity reagents can act as ligands or probes, such as lectins, aptamers, metal-organic and other substances that have affinity for other analytes [122,174,175,176,177,178,179,180,191,192,193,194,195,196,197,198,199,200,201,202,203].

**Figure 5:**
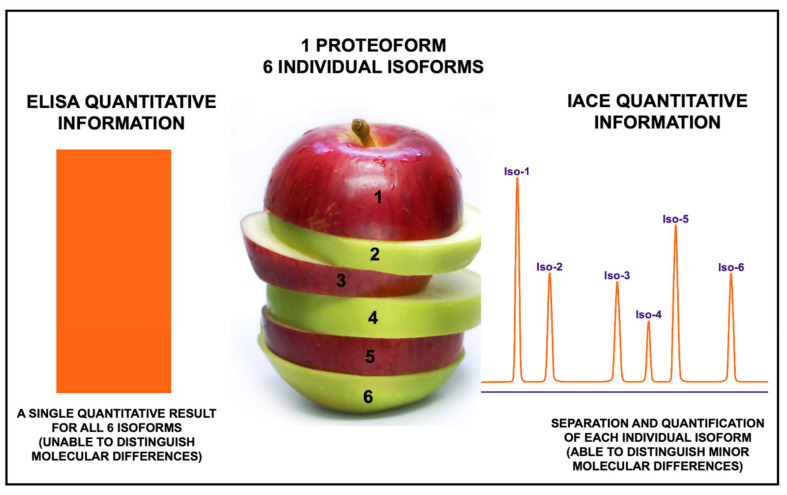
Depicts a schematic representing the main differences between the traditional mono-dimensional sandwich ELISA technique with the two-dimensional immuno-capture-separation IACE. The two-dimensional IACE technology can achieve the capture, separation, and partial characterization of each individual isoform.

In order to better represent the principle by which the two techniques, ELISA and IACE, provide qualitative and quantitative information, Figure 5 depicts an apple with 6 different slices, each having a different characteristic represented by color and size. ELISA would be able to generate information of the entire apple, and not for each individual slice. Conversely, IACE would be able to provide qualitative and quantitative data for each individual slice, represented in the figure by each modified isoform of an individual proteoform.

IACE is therefore considered to be a disruptive technique capable of discerning minor changes in substances such as metabolites of a substance or drug, or modified proteins such as proteoforms. As such, the applications of IACE are numerous, including safety regulation of new pharmaceuticals, identification of specific pathophysiologic changes that occur in inflammatory and other pathologic states, and early detection of disease through biomarker isolation and quantification. Developing biomarkers that can provide an accurate diagnosis and can predict whether patients are likely to benefit from the correct treatment is a pressing objective in many areas of medicine. IACE can be an excellent problem-solving technique, providing a new approach to monitor molecular changes throughout the evolution of a disease and through an assessment of each post-translational modification. Every modified protein component of a proteoform can change as the disease progressed, in particular the glycosylation pattern [62,200,204,205,206].

## 6. Applications of Immunoaffinity Capillary Electrophoresis. The Importance of Detecting Molecular Modifications of Proteins during the Progression of a Disease

Recombinant DNA-derived protein therapeutic products are often interchangeably referred to as biopharmaceuticals, biotherapeutics, biologicals, or biologics. Biologics are an important class of medicines, which are administered to treat an array of different diseases such as cancers, autoimmune disorders, infectious diseases, and many others [207,208,209,210,211]. The demand for biologics has risen considerable over the past few decades, owing to increase in prevalence of chronic diseases and development of plasma-derived therapies. To keep up with the demand and meet FDA and other worldwide regulatory agencies and standards for safety, production of biologics in a commercial setting requires careful control of the manufacturing processing [212,213]. This strict control necessitates a battery of sensitive analytical methodologies to both elucidate the physicochemical properties and to monitor the purity and stability of the products.

Biologics are far from ideal molecules to be used as drugs, given their potential to create toxicity and immunogenicity concerns [212,213,214]. They are often glycosylated, and their carbohydrate profiles can impact solubility, tertiary structural stability, pharmacokinetics, cellular interactions, and immunogenicity [215]. Therefore, the ability to detect subtle physicochemical differences between purified batches is therefore critical in achieving a controlled process, as well as a consistent, safe, and efficacious product [216,217,218]. This process of detecting such structural differences can be applied to a variety of pharmaceuticals that have different proteoforms, particular important glycoproteins that can affect their clinical potential and risk profiles.

**Figure 6:**
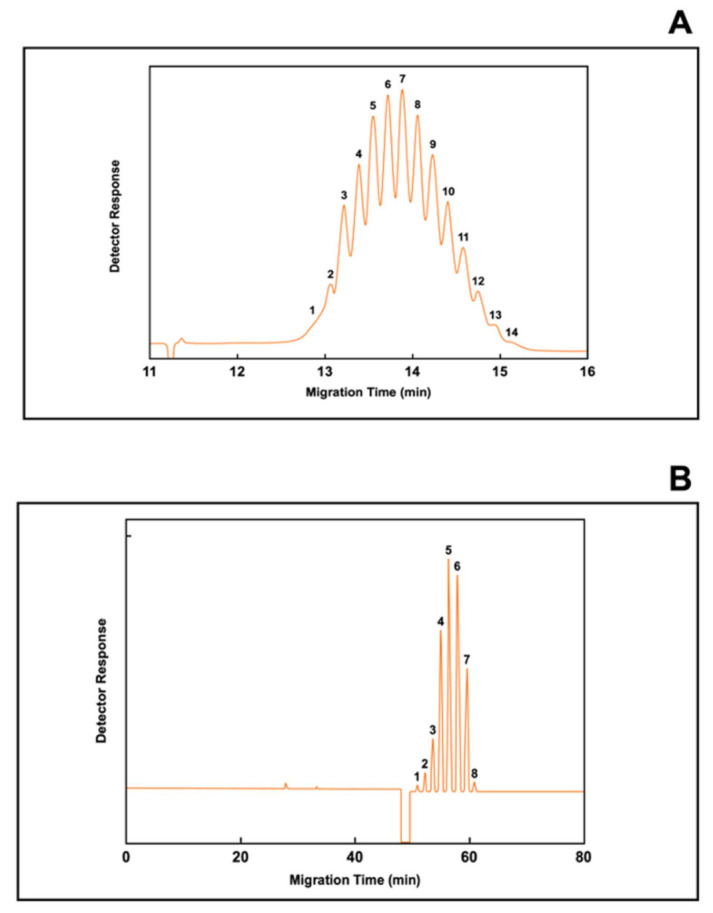
Depicts the electropherograms of 2 glycoproteins. (**A**) depicts an electropherogram of the various glycoforms of heparan-N-sulfatase separated by capillary zone electrophoresis (CZE). (**B**) depicts an electropherogram showing the various glycoforms of erythropoietin separated by CZE. Figures modified from [216,223].

Figure 6 depicts the analysis by CE of two proteoforms, heparan-N-sulfatase and erythropoietin. Heparan-N-sulfatase (HNS) is a highly glycosylated lysosomal enzyme involved in the degradation of heparan sulfate used to treat patients with mucopolysaccharidosis type IIIA or Sanfilippo syndrome type A, a rare autosomal recessive disease occurring in 1 of 10,000 live births [216]. A deficiency in HNS causes the genetic disorder. Figure 6A shows an electropherogram of heparan-N-sulfatase by capillary zone electrophoresis (CZE). At present, no treatment options is available for this neurodegenerative disorder, although the potential use of enzyme replacement therapy is currently being evaluated. Erythropoietin (EPO), also known as hematopoietin or hemopoietin, is a glycoprotein hormone produced by the kidney in response to cellular hypoxia, that promotes the formation of red blood cells by the bone marrow [219,220]. It is used to treat patients with anemia due to chronic kidney disease. Figure 6B shows an electropherogram of erythropoietin. The electropherogram represent the separation of the different isoforms of erythropoietin by capillary zone electrophoresis (CZE). Until 1990, EPO was considered to have a single biological purpose and action, the stimulation of red blood cell growth and differentiation. EPO additionally is now known to be a pleiotropic growth factor that exhibits an anti-apoptotic action on numerous cells and tissues, including malignant ones [221]. Erythropoietin is highly glycosylated. Endogenous and recombinant human EPO (rHuEPO) only differ in the composition of their N- and O-glycan moieties. The use of lectins has therefore been used to characterize subtle differences in amongst glycoproteoforms of EPO [222].

Aside from biopharmaceuticals, significant glycosylation is seen in most serum proteins and is implicated in many disease states. However, the glycome describes the complete repertoire of glycoconjugates composed of carbohydrate chains, or glycans, that are covalently linked not only to protein molecules, but also lipids [224,225,226]. More recently, it was reported that small RNAs which are modified with N-glycans are displayed on the surface of living cells [227]. Many malignancies are characterized by abnormal glycosylation, which is commonly associated with the oncogenesis and cancer progression [224,225,226,228,229,230,231,232,233,234,235,236,237,238]. Furthermore, persistent non-malignant inflammatory conditions cause continual upregulation of enzymes that promote numerous molecular changes, including glycosylation as well [228,229,230,231,232,233,234,235,236,237]. For example, differences in the PSA glycosylation pattern have been found between benign prostate alterations and prostate cancer, and between aggressive prostate cancer and indolent prostate cancer [236]. Additionally, differences in the proportions of PSA fucosylation, sialyation and GalNAc are found in aggressive stages of prostate cancer [236,237,238]. Although significant progress has been made in identifying changes in the glycosylation patterns of some proteoforms, obtaining inadequate amounts of PSA glycoforms for separation and detection has limited its clinical use in the screening and diagnosis of prostate cancer [236].

**Figure 7:**
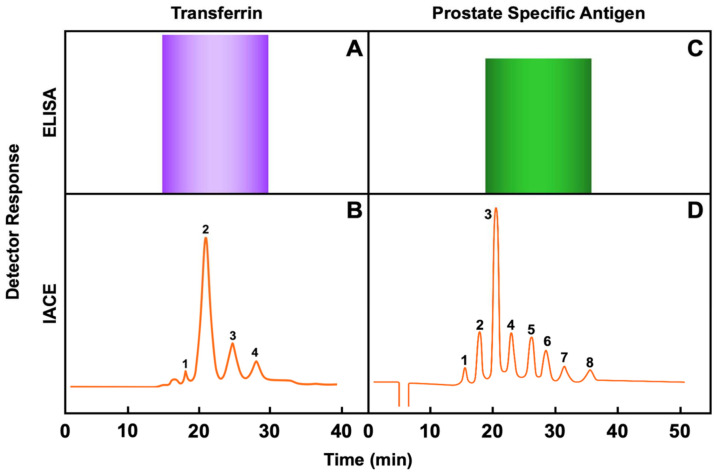
Depicts comparative results of two isoforms using ELISA, a monodimensional immunoassay, and IACE, a two-dimensional immunoassay. (**A**) depicts the analysis of transferrin using the ELISA method, and (**B**) depicts the analysis of transferrin using the IACE method. (**C**) depicts the analysis of prostate specific antigen (PSA) using the ELISA method, and (**D**) depicts the analysis of PSA using the IACE method. Figures modified from [246,247].

Figure 7 depicts comparative results of two examples of proteoforms, transferrin and prostate specific antigen, using ELISA, a mono-dimensional immunoassay, and IACE, a two-dimensional immunoassay. Figure 7A depicts the analysis of transferrin using the ELISA method, and Figure 7B depicts the analysis of transferrin using the IACE method. Transferrin (Tf) is an abundant serum metal-binding protein that delivers iron through blood circulation to cells [239,240,241]. Studies of congenital atransferrinemia in mice and humans highlight the essential role of transferrin in erythropoiesis and iron metabolism. Patients and mice deficient in transferrin exhibits anemia and a paradoxical iron overload attribute to deficiency in hepticidin, a peptide hormone synthesized largely by the liver that inhibits iron absorption and macrophage iron efflux [242]. Transferrin is also a biomarker of chronic alcohol use [243,244]. Figure 7C depicts the analysis of prostate specific antigen (PSA) using the ELISA method, and Figure 7D depicts the analysis of PSA using the IACE method. Prostate specific antigen is a glycoprotein and is expressed by both normal and neoplastic prostate tissue, making increases in PSA nonspecific [245]. However, isolation of different glycosylated forms of PSA can aid in improving the sensitivity and specificity for prostate cancer diagnosis.

**Figure 8:**
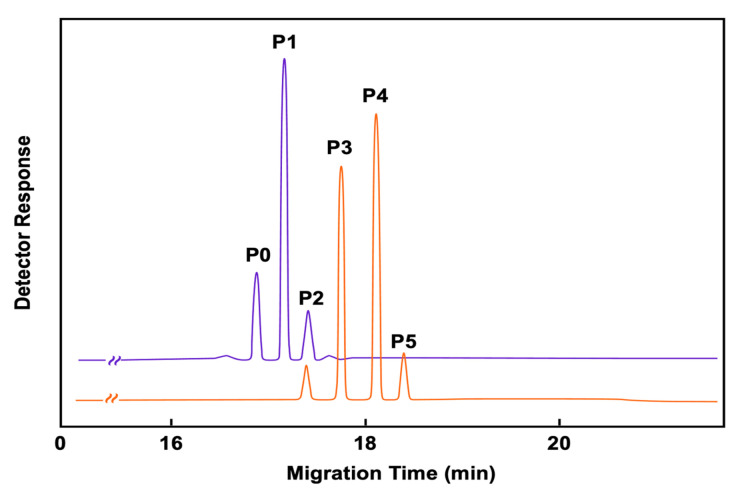
Depicts some changes occurring in an artificially created microenvironment where an intact transferrin proteoform can be altered by adding neurominidase and removing the terminal sialic acids attached to transferrin. The separation and quantification profile of the intact transferrin isoforms changes due to the removal of sialic acids which alter the mass/charge ratio of the molecules. This electropherogram was performed with an ultra-low background system that combines an advanced detector technology with signal processing algorithms. Figure modified from [248].

Improvements in the characterization of proteoforms, using an ultra-low background system that combines an advanced detector technology with signal processing algorithms, have provided various sialoform profiles of transferrin. For example, as depicted in Figure 8, two aliquots of transferrin subjected to sialic acid cleavage were separated by capillary zone electrophoresis [248]. In the first aliquot, transferrin was incubated for 1 h with neuraminidase, and the resulting electropherogram shows primarily 3 isoforms, P0 (asialo-Tf), P1 (monosialo-Tf), and P2 (disialo-Tf). The second aliquot, transferrin was incubated for 5 h with neuraminidase, and the resulting electropherogram shows 4 isoforms, but changes occur in migration times and quantification. The resulting electropherogram shows P2, P3 (trisialo-Tf), P4 (tetrasialo-Tf) and P5 (pentasialo-Tf). Variation in serum sialoform content can be correlated to different pathological states, such as carbohydrate-deficient transferrin (CDT) [249], congenital disorders of glycosylation (CDG) [250], and cancer [251]. These improvements having better data acquisition, in combination with IACE, might result in superior data reproducibility to monitor minor protein changes during the course of progression of a disease.

As depicted in the above figures, IACE has the capability of capturing, separating and detecting glycoproteins, including those present in minute quantities. The target proteoform is captured and bound to antibodies immobilized to a solid support within a portion of the capillary or microchip channel, or directly into the wall of the capillary or channel. If enough protein substance is bound to the antibody, it is then eluted and detected on-line usually by ultraviolet detection. Alternatively, if not enough protein substance is bound, the protein can be labeled in situ by a fluorescent chromophore, separated by one or more of the CE separation modes, and detected on-line using a laser-induced fluorescence. Ideally, labeling and preconcentrating of a protein sample prior to introducing the sample into the capillary, will enhance even further the detection of a protein found at low abundance in complex mixtures. Furthermore, the CE instrument can be coupled to a mass spectrometer providing additional protein information. Peptide mapping of the proteoform can also be carried out, as well as identification of the sugar moieties of the proteoform [123,151,174,197,198,199,200,201,202,203,246,252,253]. Greater implementation of IACE therefore has the potential to further understand protein biomarkers, their application to disease states and pharmaceuticals, and have the potential to improve health outcomes through prevention and early diagnosis of disease.

## 7. Future of Immunoaffinity Capillary Electrophoresis for the Analysis of Proteoforms

With the growing geriatric population and the increasing prevalence of chronic diseases across the globe, growing demand for improving diagnostic laboratory techniques will continue. Proteomics should ideally be the driver for improving diagnostics and will serve as a primary method for detection of molecular changes indicative of disease states. Numerous laboratories have reported a plethora of assays using capillary electrophoresis in conventional format and in microfluidic format for detection and quantification of several proteoforms [254,255,256,257,258,259,260,261,262,263,264,265,266,267]. However, in order for proteoforms to reach their full clinical potential, it is necessary to be able to accurately detect and quantify near all proteoforms in biological fluids using advanced and sophisticated analytical instrumentation, such as IACE.

Use of immunoaffinity capillary electrophoresis in conventional CE instruments or in a point-of-care instrument has a significant potential to profile proteoform patterns in biological fluids, and in proteins obtained from circulating tumor cells, circulating immunological cells, exosomes, and other cellular or vesicles present in biofluids [123,174,175,195,196,197,198,199,200,201,202,203,246,247]. The ideal proteoform biomarker would be cost-effective, objective, fast to process and easy to interpret, with high clinical diagnostic sensitivity and specificity. IACE can capture and separate one or more proteoforms simultaneously, with the capability to discern minor molecular changes at high analytical sensitivity and specificity, and the data can be obtained in a short period of time with higher accuracy than conventional immunoassays. Furthermore, IACE is a more sustainable and affordable technology, given that it minimizes its use of samples and reagents. Ideally, it can also be used in tandem with telemedicine, to further improve access to and cost of healthcare.

The future of the 21st century medicine will include the use of POCT instruments, allowing home-based monitoring of wellness and disease using small samples of biological fluids for liquid biopsy. This will incorporate conveniently obtained specimens such as oral fluid, sputum, urine, sweat and tears. In summary, IACE technology will shorten the clinical decision path, offering a quick and accurate means of detecting disease and selecting the best treatment for a particular illness without compromising safety or effectiveness.

## Figures and Tables

**Table 1:** Association of Proteoforms with Physiological Functions and Diseases.

Proteoform	Function	Clinical Significance	Reference
Cystatin C	Endogenous cysteine protease inhibitor.	Assessment of progression of kidney function. Increased levels of Cystatin C can be found in acute and chronic kidney disease.	[48]
Chemokine RANTES	Key role in inflammation, cell recruitment, and T cell activation.	Associated with autoimmune diseases, arthritis, diabetes, obesity, cardiovascular diseases, breast and cervical cancers, and other diseases.	[49]
Amyloid proteoforms	Complication of normally soluble proteins that can join together to form an insoluble protein.	Associated with infiltrative proteinopathies, including Alzheimer’s disease, restrictive cardiomyopathy, infiltrative kidney disease, and other diseases.	[50,51]
Amyloid-beta (A*β*) proteoforms	Amyloid-beta (A*β*) plays a key role in the pathogenesis of Alzheimer’s disease. Post-mortem identification of 26 proteoforms in the brain of Alzheimer’s disease patients has provided significant information in patients suffering from Alzheimer’s disease (AD) and dementia.	The heterogeneity of A*β* proteoforms deepens our understanding of Alzheimer’s disease and offers new avenues for investigation into pathological mechanisms of the disease, with implications for therapeutic development.	[52]
Lacritin proteoforms	Prevent tear film collapse and maintain epithelial homeostasis.Proteoforms include an active monomer, inactive polymers, and a splice variant termed lacritrin-c.	Associated with dry eye disease. Important in visual acuity and promotes basal tearing.Quantitation of the different proteoforms of tear lacritin may provide a diagnostic tool for ocular diseases.	[53,54]
Tumor suppressor PTEN	Control various aspects of cellular function, grouping them into three categories: intrinsic, function-induced, and inducible proteoforms.	PTEN proteoforms offer novel therapeutic opportunities in the treatment of various cancers and other diseases.	[55]
Cardiac troponin	Critical regulator of cardiac muscle contraction and relaxation.	Assessment of cardiac troponin proteoforms in serum of patients with acute myocardial infarction and other forms of myocardial injury.	[56,57]
MM-9 proteoforms	Matrix metalloproteinases (MMPs) are a class of secreted or cell bound endopeptidases, implicated in every step of the process of inflammatory cell migration.	Specific inhibition of MMPs has been suggested to be an interesting approach to control inflammation.	[58]
Apolipoprotein(a)	Lipoprotein(a) (Lp(a)) is an LDL-like particle, that contains a single copy of the apolipoprotein(a) covalently linked by a disulfide bridge to apolipoprotein(b). Its function is in wound healing, where it promotes tissue repair and vascular remodeling.	Lipoprotein(a) plays a role as an independent risk factor in the development of atherosclerotic cardiovascular diseases and calcified aortic valve disease. New research indicates that Lp(a) should be evaluated in terms of its apo(a) component and no longer in terms of Lp(a) mass.	[59]
Apolipoprotein A-1	Apolipoprotein A1 (Apo A-1) is the major constituent of human high-density lipoproteins, which plays a key role in reverse cholesterol transport and lipid homeostasis.	Apo A-1 exhibits antioxidant and anti-inflammatory properties and inhibits the aggregation and neurotoxicity of amyloid-beta peptide in Alzheimer’s disease. Apo A-1 may possibly provide protection against neurological disorders.	[60]
DJ-1 proteoforms	DJ-1 is a cancer associated protein that protects cells from oxidative stress, functioning as a deglycase enzyme. DJ-1 acts as a redox-sensitive chaperone and as an oxidative stress sensor.	Associated with breast cancer. The modulation of specific DJ-1 function might produce substantial anticancer effects.	[61]
Prostate specific antigen proteoforms	Prostate specific antigen (PSA) is a glycoprotein with protease activity. This androgen-regulated serine-protease participates in the dissolution of the seminal fluid coagulum and plays an important role in fertility. PSA is produced by both prostate epithelial cells and prostate cancer cells.	Commonly used as serum biomarker for prostate cancer. Analysis of PSA proteoforms in urine and assessment of intact protein and glycopeptide analysis might be useful in improving prostate cancer screening.	[62]
Haptoglobin proteoforms	Serum haptoglobin (Hp) is a glycoprotein that scavenges freely circulating hemoglobin leaked into the blood stream when erythrocytes are damaged or die. Hp possesses four N-glycosylation sites on the beta-chain.	Studies have shown differences in the glycosylation pattern among patients with liver cirrhosis and hepatocellular carcinoma.	[63]
Erythropoietin	Erythropoietin (EPO) is a glycoprotein hormone of significant importance in the formation of red blood cells, as well as in other physiological functions.	EPO is used as a therapeutic protein for the treatment of anemia in chronic kidney disease and cancer.	[64,65,66]
Kidney allograft proteoforms	Proteins identified in peripheral blood mononuclear cells (PBMCs) as molecular signatures of kidney allograft pathology.	Non-invasive differential diagnostics of dysfunction of a transplanted kidney, or biomarkers of the kidney graft rejection.	[67]
EgAg B proteoforms	EgAg B proteoforms are parasite antigen proteins present in cystic echinococcocis disease caused by the Echininococcus granulosus metacestode parasite. These antigens are immunopotent.	Biomarkers for early detection and monitoring of the progression of the cystic echinococcosis disease. Specific immunodominant epitopes change as the disease progresses.	[68]
Human growth hormone proteoforms	Human growth hormone (hGH) is synthesized by, stored in, and secreted by the pituitary gland. It promotes human growth and metabolism.	Monitoring of the proteoform pattern changes in a growth hormone-secreting pituitary adenoma, when compared to control pituitary tissues, can be of significant value for the predictive diagnosis, targeted prevention, and treatment of pituitary adenoma.	[69]
∆Np73 proteoforms	The p53 family of proteins, including p53, p63 and p73, have a role in tumor suppression. The ∆Np63 and ∆Np73 proteoforms are frequently overexpressed in a wide range of tumors, where they are associated with poorer prognosis. Furthermore, it has been demonstrated that the presence of autoantibodies to p53, p63 and p73 proteins exists in the serum of cancer patients.	∆Np73 proteoforms shows a specific seroreactivity different from that of p73 with a higher diagnostic ability to discriminate between colorectal cancer patients and controls, and especially premalignant individuals and controls which may have an important impact on cancer prevention to predict premalignant tumors.	[70]
Surfactant protein B immature proteoform (proSP-B)	Surfactant protein B (SP-B) is a protein vital for normal lung function. Higher levels of circulating SP-B have been found in heavy smokers. Immature SP-B (proSP-B) flow into the bloodstream, where it binds high-density lipoprotein (HDL), modifying its function. Impairing the alveolar cell SP-B metabolism is likely the trigger of the smoke-induced pro-atherosclerotic cascade.	Circulating immature proteoform of surfactant protein B (proSP-B) has been proposed as the most reliable lung-specific marker for alveolar-capillary membrane dysfunction and overall clinical status of heart failure.	[71]
Sarcomeric proteoforms	The post-translational modifications (PTMs) of sarcomeric proteins are known to be important mediators of cardiac signaling and exert various effects on contractile function. Hypertrophic cardiomyopathy (HCM) is the most common inherited disease and a leading cause of sudden death in young adults. It is characterized by abnormal thickening of the myocardium, which imposes a mechanical burden on the heart. HCM is highly heterogeneous and has been linked to mutations in the genes that encode proteins of the sarcomere.	Obtaining a comprehensive view of the changes in the sarcomeric proteome is an important first step toward understanding the molecular underpinnings of HCM. Future proteomics studies covering a wide range of HCM phenotypes will hold promise to help define disease progression and prognosis based on the proteoform landscape.	[72]
NISTmAB proteoform	The NISTmAb is a recombinant humanized monoclonal antibody Reference Material from the National Institute of Standards and Technology. It is a class representative IgG1κ intended to serve as a pre-competitive platform for harmonization and technology development in the biopharmaceutical industry.	Complex biotherapeutics, in particular monoclonal antibodies, increasingly dominate the arena of new drugs submitted for regulatory approval. Quality control mechanisms and their accompanying analytics are still evolving to meet increasingly sophisticated needs of the biotherapeutics market. Deviations in quality control may be linked to pathological conditions, such as immunogenicity or toxicity.	[73]
Natriuretic peptides proteoforms	The natriuretic peptide family consists of three biologically active peptides: atrial natriuretic peptide (ANP), brain (or B-type) natriuretic peptide (BNP), and C-type natriuretic peptide (CNP). Among these, ANP and BNP are secreted by the heart and act as cardiac hormones. ProANP and B-ANP are minor forms in the circulation but increases in patients with heart failure.	The human BNP precursor proBNP is proteolytically processed to BNP_1–32_ and N-terminal proBNP (NT-proBNP) within ventricular myocytes. Uncleaved proBNP as well as mature BNP_1–32_ and NT-proBNP are secreted from the heart, and its secretion is increased in patients with heart failure. Furthermore, NT-proBNP is O-glycosylated in the plasma of patients with severe heart failure.	[74,75]
Transthyretin proteoforms	Transthyrein (TTR) is a serum and cerebrospinal fluid (CSF) protein with metalloprotease activity. It is one of the most abundant proteins in CSF. Despite being best known for transporting thyroxine (T4) and retinol through the blood–brain barrier (BBB), TTR has been suggested to play a role in a broad range of functions in the central nervous system.	Dysregulations of TTR levels characterize several neurological disorders. In patients with stroke, the detection of TTR has been described as a positive prognostic indicator of clinical outcomes. TTR proteoforms are changed in the CSF of patients affected with spinal muscular atrophy (SMA) type, after treatment with the antisense oligonucleotide nusinersen.	[76]
C-reactive protein proteoforms	C-reactive protein (CRP) is an acute inflammatory protein that increases up to 1,000-fold at sites of infection or inflammation. CRP is produced as a homopentameric protein, termed native CRP (nCRP), which can irreversibly dissociate at sites of inflammation and infection into five monomers, termed monomeric CRP (mCRP). CRP is synthesized primarily in liver hepatocytes but also by smooth muscle cells, macrophages, endothelial cells, lymphocytes, and adipocytes. Having been traditionally utilized as a marker of infection and cardiovascular events, there is now growing evidence that CRP plays important roles in inflammatory processes and host responses to infection including the complement pathway, apoptosis, phagocytosis, niric oxide (NO) release, and the production of cytokines, particularly interleukin-6 and tumor necrosis factor-α.	CRP isoforms have distinct biological properties, with nCRP often having more anti-inflammatory activities compared to mCRP. The nCRP isoform activates the classical complement pathway, induces phagocytosis, and promotes apoptosis. On the other hand, mCRP promotes the chemotaxis and recruitment of circulating leukocytes to areas of inflammation and can delay apoptosis. mCRP increases interleuin-8 and monocyte chemoattractant protein-1 production.	[77]
SHC-Transforming protein 1 proteoforms	Human SHC-Transforming protein 1 (Shc1) has been found to be important in the regulation of apoptosis and drug resistance in mammalian cells. Shc1 is an intracellular scaffold protein, which is involved in downstream pathways of cell surface signaling receptors, such as the insulin signaling pathway. One of the isoforms of Shc1 is p66shc, a mitochondrial associated oxidative stress biomarker, that may be involved in regulating the life span and the effects of reactive oxygen species (ROS). P66shc has been implicated in several metabolic pathways, being able to act as an adaptor protein as well. Furthermore, p66shc is significantly altered in patients with elevated blood glucose levels.	Evaluation of p66shc/Shc1 has been reported to be a useful addition to the regularly used biomarkers, such as HbA1c, to increase diagnostic sensitivity for the identification of prediabetes (T2DM). Knowledge of the presence of oxidative stress and inflammatory processes, forming a complex pattern of disease progression, is therefore an important step towards early, effective treatment.	[78,79]
Prolyl hydroxylase alpha subunit proteoforms	Mammalian prolyl 4-hydroxylase (P4H) is a tetramer composed of two unique subunits, alpha and beta. One gene makes the beta subunit that functions independently as a protein disulfide isomerase, and the other genes make three alpha subunit isoforms.	P4H catalyzes selective proline-containing peptides to hydroxy-proline-containing peptides. The best-known role of hydroxyproline is in stabilizing the collagen triple helix. A prolyl hydroxylase domain protein acts on the hypoxia inducible factor alpha subunits, which plays a key role in sensing molecular oxygen. Prolyl hydroxylases are essential for breast cancer metastasis, and a prolyl hydroxylase inhibitor decreases tumorogenesis.	[80,81,82,83,84,85,86,87,88]
Immunoglobulin G (IgG) proteoforms	Beyond their ability to neutralize pathogens, antibodies can mediate an array of effector functions through their interactions with Fc-receptors, complement molecules, and mammalian lectin-like molecules. Immunoglobulin G (IgG) fragment antigen binding (Fab) region binds a specific antigen, while its fragment crystallizable (Fc) region binds different receptors on the surface of various immune cells, thereby dictating the type of immune response elicited by the antigen binding.	Analysis of Fc-specific IgG glycosylation is critical for population-level studies of how antibodies may vary in response to vaccination or infection, and across disease states ranging from autoimmunity to cancer in both clinical and animal studies. IgG glycans are an excellent biomarker of biological age. Sialylation of Fc glycan has been reported to have anti-inflammatory activity.	[89,90,91]
Histone proteoforms	Chromatin is the structural framework that packages DNA into chromosomes within the nucleus of a cell. Histones comprises the principal protein component of chromatin and are involved in the regulation of gene expression. This epigenetic configuration is achieved through complex patterns of post-translational modifications, the incorporation of histones variants, and through controlled histone proteolysis.	Histone post-translational modifications (PTMs) are one of the main mechanisms of epigenetic regulation. Dysregulation of histones PTMs leads to many diseases, such as cancer.	[92,93,94,95]
KRAS proteoforms	Mutations of the KRAS gene are found in human cancers with high frequency and result in the constitutive activation of its protein products.	Mutations affecting post-translational modifications leads to aberrant regulation of downstream pathways, promoting cell survival, proliferation, and tumorigenesis that drive cancer progression and negatively affect treatment outcomes.	[96]
Huntingtin proteoforms	Huntington’s disease (HD) is a rare neurodegenerative disorder caused by the aberrant expression of mutant Huntingtin (HTT) protein containing an expanded polyglutamine tract. The expression of this protein is highly enriched in the brain relative to other tissues with highest expression in neurons.	HTT is now known to have a wide variety of post-translational modifications, including phosphorylation, sumoylation, acetylation, ubiquitination, and protease cleavage. Correlating these HTT proteoforms with functions should be fruitful in identifying mechanism of pathology that can be targeted for intervention.	[97]
Alpha-synuclein proteoforms	Cumulative evidence suggests that lysosomal dysfunction contributes to neurodegenerative diseases, especially if amyloid proteins are involved. Among these, alpha-synuclein that progressively accumulates and aggregates in Lewis bodies is undisputedly a main culprit in Parkinson disease.	Alpha-synuclein can possess diverse post-translational modifications, aggregate formations, and truncations, all of which contribute to a growing set of proteoforms. These interfere directly or indirectly with lysosome function, reducing their own degradation, and thereby accelerating the protein aggregation and disease process.	[98]
Carbonic anhydrases proteoforms	Metalloenzymes carbonic anhydrases intervene in the second and rate limiting step of the catalytic mechanism of carbon dioxide reversible hydration. Genetic deficiencies of several carbonic anhydrases have been reported to be associated with diseases such as osteopetrosis, cerebral calcifications, hyperammonemia, retinal problems, and hyperchlorhydrolysis.	The loss of function of carbonic anhydrases would be in principle be treatable with selective activators of these enzymes. Carbonic anhydrase proteoforms represent a crucial family of new targets for improving cognition, but also in therapeutic, such as phobias, obsessive compulsive disorder, generalized anxiety, and post-traumatic disorders, for which few therapies are available. Studies on carbonic anhydrase proteoforms are important to elucidate the role of these modified proteins and their potential activators in brain processes.	[99]

## Data Availability

The data is available, upon request due to privacy/ethical restrictions, directly from the corresponding author of the publication where the information was obtained. Alternatively, the data is openly available in a public repository that does not issue DOIs.
